# 20 Years with SGBS cells - a versatile in vitro model of human adipocyte biology

**DOI:** 10.1038/s41366-022-01199-9

**Published:** 2022-08-19

**Authors:** Daniel Tews, Rolf E. Brenner, Reiner Siebert, Klaus-Michael Debatin, Pamela Fischer-Posovszky, Martin Wabitsch

**Affiliations:** 1 Department of Pediatrics and Adolescent Medicine, Division of Pediatric Endocrinology and Diabetes, Ulm University Medical Center Ulm, Ulm, Germany; 2 Department of Orthopaedics, Division for Biochemistry of Joint and Connective Tissue Diseases University of Ulm, Ulm, Germany; 3 Institute of Human Genetics, University of Ulm and Ulm University Medical Center, Ulm, Germany

**Keywords:** Obesity, Cell biology

## Abstract

20 years ago, we described a human cell strain derived from subcutaneous adipose tissue of an infant supposed to have Simpson-Golabi-Behmel Syndrome (SGBS), thus called “SGBS cells”. Since then, these cells have emerged as the most commonly used cell model for human adipogenesis and human adipocyte biology. Although these adipocyte derived stem cells have not been genetically manipulated for transformation or immortalization, SGBS cells retain their capacity to proliferate and to differentiate into adipocytes for more than 50 population doublings, providing an almost unlimited source of human adipocyte progenitor cells. Original data obtained with SGBS cells led to more than 200 peer reviewed publications comprising investigations on adipogenesis and browning, insulin sensitivity, inflammatory response, adipokine production, as well as co-culture models and cell-cell communication. In this article, we provide an update on the characterization of SGBS cells, present basic methods for their application and summarize results of a systematic literature search on original data obtained with this cell strain.

## Introduction

**Fig. 1:**
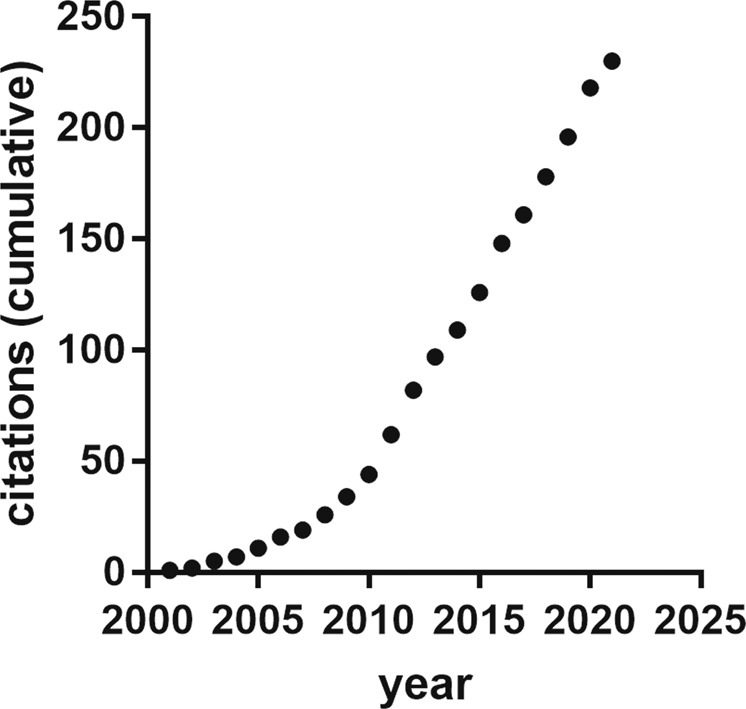
Original articles publishing data obtained with SGBS cells. Cumulative number of publications from 2001 to 2021 which contain data obtained using SGBS cells.

In vitro models of adipocytes are crucial to understand molecular mechanisms of adipogenesis, the development of insulin sensitivity, the pathogenesis of insulin resistance, and the regulation of adipokine expression and secretion. To date, cells of murine origin have been frequently used to study adipogenesis as well as the metabolic and endocrine function of adipocytes. Moreover, conventional primary human adipose tissue-derived stromal cells have been used to gain more physiological relevant data. However, these cells undergo cellular senescence providing only a limited source of material. Few human adipocyte cell models exist but are either transformed or are generated from human tumor material (see Table 1). So-called “Simpson-Golabi-Behmel Syndrome (SGBS) cells” (RRID: CVCL_GS28) derive from an infant with clinical features suggesting the eponymous syndrome, and are characterized by prolonged proliferation and differentiation capacity. Today, these cells have become the most commonly used human cellular model to study insulin sensitivity and adipocyte metabolism - key aspects for understanding pathophysiology of type 2 diabetes and obesity - under chemically defined conditions. Since their introduction in 2001 [1], the cells have been used in various studies resulting in more than 200 original publications (Fig. 1).

**Table 1:** In vitro model systems for adipogenesis.

Name	Reference	Species	Origin	Potency	Status	Transformed
3T3-L1	Green [92]	mouse	swiss albino mouse embryo, L1 subclone	uni	hypertriploid	No
3T3-F442A	Green [93]	mouse	swiss albino mouse embryo, F442A subclone	uni	hypertriploid	No
MS-5	Itoh [94]	mouse	irradiated bone marrow stromal cells		hypertriploid	X-ray
OP9	Gao [95]	mouse	bone (calvaria)	multi		No
WT-1	Tseng [96]	mouse	brown adipose tissue stromal cells	uni (BAT)		SV40
T37i	Zennaro [97]	mouse	murine hibernoma	uni (BAT)		SV40
Ob17	Negrel [98]	mouse	de-differentiated murine white adipocytes			No
C3H/10T1/2	Pinney [99]	mouse	C3H mouse embryo	multi		No
HIB1B	Ross [100]	mouse	murine hibernoma	uni (BAT)		SV40
hMADS	Rodriguez [101]	human	sc adipose tissue	multi	diploid	no
DFAT	Matsumoto [102]	human	sc adipose tissue	multi	diploid	no
LipPD1	Kässner [103]	human	lipoma	uni		no
PAZ6	Zilberfarb [104]	human	brown adipose tissue	uni		SV40
LiSa-2	Wabitsch [105]	human	liposarcoma	uni	hypertriploid	no
SGBS	Wabitsch [1]	human	sc AT from infant diagnosed with SGBS	multi	diploid	no

In this review, we comment on the origin of the cells and systematically describe their adipogenic differentiation and features of cellular metabolism (Part A). In the main body (Part B) we present results of a systematic literature analysis on original data obtained with SGBS cells as well as on methods developed and applied with this cell strain.

## Part A: Origin of SBGS cells and systematic description of their adipogenic differentiation and metabolic characterization

### SGBS origin

SGBS cells have been isolated from subcutaneous adipose tissue of an infant with clinically suspected diagnosis of Simpson-Golabi-Behmel syndrome (SGBS) [2]. Phenotypic findings in the male patient have been diffuse neonatal hemangiomatosis, fetal overgrowth, a prominent forehead, a short, broad upturned nose, postaxial hexadactyly of both hands and the right foot, six lumbar vertebrae, as well as hydramnios [2]. SGBS type 1 (OMIM: 312870) is a rare, X-linked inherited disorder characterized by pre- and postnatal overgrowth, based on genetic rearrangements or point mutations involving the glypican 3 (GPC3) gene [3–5], occasionally also including the glypican 4 (GPC4) gene [6]. Another infrequent clinical subtype, SGBS type 2 (OMIM: 300209) is based on a genetic variant in the OFD1 (Outer Dense Fiber Of Sperm Tails 1) gene [7]. Despite the clinical features of the patient, no variants in the GPC3- as well as GPC4- and OFD1-genes could be confirmed, so that the molecular cause of the underlying disease remains uncertain.

### Adipogenic differentiation and metabolic characterization

A protocol for adipogenic differentiation of SGBS cells has been published earlier [1]. A detailed protocol is found in the Methods section. In the following paragraphs, we show new data on adipogenic differentiation and present them in a systematic manner in order to complete the original publication [1].

**Fig. 2:**
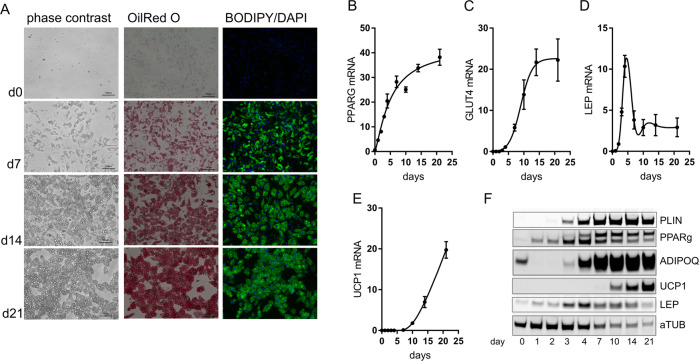
Adipogenic differentiation of SGBS cells. SGBS preadipocytes were cultured in differentiation medium for up to 21 days. **A** Cells were fixed and intracellular lipids were stained using either OilRed O or BODIPY, bar = 100 µm. **B**–**F** During the time-course of adipogenic differentiation, expression of key adipogenic marker genes were determined using qRT-PCR (**B**–**E**) or Western Blot (**F**). Data + SEM of *n* = 3 experiments are shown, (**F**) one representative blot is shown.

Using serum-free, defined conditions, SGBS cells develop from fibroblastoid cells into adipocytes containing multiple lipid droplets, which can be visualized by commonly used lipid stains such as OilRed O or BODIPY (Fig. 2A). Fourteen days after induction of adipogenic differentiation, lipid droplets start to fuse. During long-term cultures cells with large lipid droplets tend to detach from the plastic surface SGBS cells express common markers of adipogenesis during the time-course of differentiation, including PPARG and GLUT4 (Fig. 2B). Leptin mRNA as well as protein is induced early in differentiation due to the presence of dexamethasone during the first 4 days of the protocol, and remains stable at a lower level (Fig. 2D, F). Interestingly, uncoupling protein 1 (UCP1) the marker of brown adipose tissue, is induced during adipogenesis as well due to a browning effect of the PPARG agonist rosiglitazone (Fig. 2E, F) [8]. Studies investigating these effects are discussed further below.

The remarkable feature of these cells is certainly that their ability for adipogenic differentiation is preserved for up to 50 population doublings [1], although the cells are not manipulated for transformation of immortalization. This is in contrast to primary ASCs obtained from other infants in which adipogenic differentiation capacity regresses rapidly [1].

**Fig. 3:**
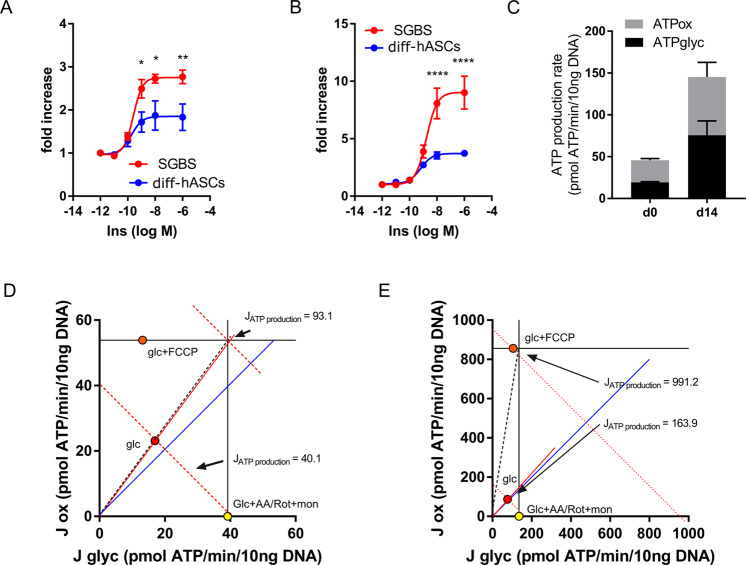
Metabolic properties of SGBS preadipocytes and adipocytes. **A** Insulin-dependent ^14^C-2-deoxyglucose uptake and (**B**) glucose incorporation into lipids was determined in SGBS cells and primary human adipose-derived stromal cells (diff-hASCs) differentiated for 14 days into adipocytes in vitro. Data +SEM of *n* = 4–5 experiments are shown, *p < 0.05, **p < 0.01, ****p < 0.0001 (ANOVA). **C**–**E** Oxidative and glycolytic activity was determined in SGBS before (d0) and after (d14) adipogenic differentiation. ATP production rates (**C**, *n* = 2) were determined from the corresponding oxygen consumption and extracellular acidification rates. Bioenergetic plots of preadipocytes (**D**) and adipocytes (**E**) were calculated using metabolic activators and inducers. Data are normalized for DNA content, one representative experiment of two is shown. Glc Glucose, FCCP Carbonyl cyanide-p-trifluoromethoxyphenylhydrazone, AA Antimycin A Rot rotenone, mon monensin.

A specific feature of differentiated SGBS adipocytes is their pronounced insulin sensitivity. Upon starvation from differentiation factors for 24 h and re-stimulation with increasing concentrations of recombinant insulin, cellular uptake of ^14^C-2-desoxyglucose increases dose-dependently up to approx. 3-fold with an EC_50_ of 250 pM. For comparison, we determined glucose uptake in in vitro differentiated adipocytes from adipose stromal cells (ASCs) obtained from infants (age 0 to 5 months), in which maximal uptake was significantly lower, however with comparable EC_50_ values of 180pM (Fig. 3A). Similar patterns were found when we investigated insulin-dependent *de novo* lipogenesis by measuring the incorporation of ^14^C- glucose into lipids (Fig. 3B). Upon stimulation with insulin, glucose incorporation increased by approx. 9-fold, while the increase in adipocytes differentiated from control cells was approx. 4-fold.

Determination of cellular bioenergetics and ATP generation is of particular interest in the obesity field, and introduction of plate-based respirometers has simplified the measurement of energy fluxes in different cell types. Using a Seahorse XF Bioanalyzer, we studied oxidative and glycolytic ATP production rates in SGBS cells in the preadipocytes and adipocyte state (Fig. 3C–E). In the basal state, ATP demand in preadipocytes is met by both oxidative as well as glycolytic ATP generation (Fig. 3C). In adipocytes, ATP generation increases severalfold, indicating a higher basal energy demand compared to preadipocytes (Fig. 3C). Using common mitochondrial inhibitors and activators, we interrogated standard parameters of mitochondrial and glycolytic activity, calculated ATP production rates and displayed those as bioenergetic plots as described elsewhere (Fig. 3D, E) [9]. When fully stimulated, SGBS preadipocytes can almost double their ATP production rate from 40 to 90 pmol ATP/min/10 ng DNA. In adipocytes, this increase is far higher (5.5-fold, 900 vs 160 pmol ATP/min/10 ng DNA), reflecting an increased ATP demand in differentiated adipocytes to meet cell-specific functions. Interestingly, this demand is mainly fed by oxidative ATP production, revealing a large oxidative reserve capacity in adipocytes.

As depicted below, measurements of cellular bioenergetics have been frequently applied in SGBS cells, especially in the field of adipocyte browning.

## Part B: SGBS cells serve as model system in several research areas – results of a systematic literature search

We performed a systematic literature search in PubMed (https://pubmed.ncbi.nlm.nih.gov/) using the term “SGBS cells” and “SGBS adipocytes” from the date of the original publication in 2001 until Dec. 31^st^, 2021. All entries were then manually curated and only those publications containing data using SGBS cells were taken. The full list of 230 publications is provided in [Supplementary data]. Due to space restraints, only 76 publications were referred to in this review. These publications have been selected according to the research fields we aimed to cover in this manuscript.

Obesity is characterized by an abnormal accumulation of adipose tissue causing metabolic disease. This accumulation is accompanied by a low-grade inflammation of the tissue, leading to alterations in adipokine secretion, and induction of insulin resistance in the adipocytes. Recently, the transition of white adipocytes to a brown adipogenic program (referred as to “browning”) has gained substantial interest in the obesity field. In the following paragraphs we summarize data obtained with SGBS cells in these research areas as well as methods related to their use in the respective context.

### SGBS cells in comparison to other cell models of adipogenesis

In the literature, data of direct comparisons of SGBS cells with other cell models of adipogenesis is limited. In our original publication we demonstrated that, in contrast to primary isolated adipose stromal cells (ASCs), SGBS retain their ability to undergo adipogenic differentiation after prolonged serial passaging, and express common markers of adipogenesis [1]. Moreover, cell models differed in terms of growth rate, with SGBS cells having a slightly higher doubling time [1].

In a recent publication, we compared SGBS cells with primary ASCs regarding their ability to undergo adipocyte browning [8]. We could show that, under identical culture conditions, expression of common adipogenic marker genes were comparable, but expression of UCP1 was higher in SGBS cells, whereas mitochondrial content was lower compared to adipocytes differentiated from primary ASCs. As depicted above, SGBS cells and primary ASCs from children were similar in terms of insulin sensitivity, but maximal glucose uptake was higher in SGBS cells (Fig. 3).

Another frequently used human adipocyte model are so-called hMADS (human multipotent adipose-derived stem cells) [10]. We could recently demonstrate that these cells behave similar in terms of regulation of adipogenesis in response to inhibition of specific miRNAs [11] as well as in browning upon TGFβ2 signaling activation [12].

The most commonly used adipocyte model is murine 3T3-L1 fibroblasts (RRID: CVCL_0123). In a recent publication, Schmidt et al., mapped binding sites of PPARγ and C/EPBα in SGBS and murine 3T3-L1 cells [13]. Although the conservation of the overall regulatory regime and putative target genes between mouse and human adipocytes is high, they demonstrate that the retention of mouse binding sites in human is limited, i.e., most sites are species-specific. In another study, Rossi et al. demonstrated that, upon induction of insulin resistance by chronic insulin treatment, 3T3 cells showed a strong decrease in insulin-stimulated glucose uptake, while SGBS cells showed only a minor impairment. Moreover, they found differences in gene expression upon chronic insulin treatment between SGBS and 3T3-L1 cells. For example, leptin expression was significantly increases in 3T3 cells, while in SGBS cells leptin mRNA levels were stable upon chronic insulin treatment [14] further suggesting differences in murine and human SGBS adipocytes.

### Regulation of adipogenesis

Development of pharmacologic therapies in obesity and insulin resistance treatment requires reliant human cell models to test their effect on adipogenic differentiation. Moreover, investigation of genes potentially affecting adipogenesis needs cells which can be genetically modified easily.

SGBS cells have been used to understand basic principles of adipogenesis, e.g., by comparing transcription factor usage in human and rodent cells [13], or by investigating the effect of radiation [15] or compressive force on human preadipocytes [16]. Using gain- and loss-of-function experiments, the effects of different [17–20] regulators of adipogenesis have been addressed. Recent literature demonstrates that microRNAs play a pivotal role in adipogenesis. Some of these, such as miR-130a, miR-375 [21, 22], miR-192* [23], and miR-27a [24] have been investigated in SGBS cells.

Weight loss/lipodystrophy in HIV patients has been attributed to adverse effects of anti-retroviral therapy. It has been demonstrated by us [25] and others [26] that certain HIV protease inhibitors inhibit adipogenic differentiation in SGBS cells.

As discussed below, obesity is characterized by alterations of the adipose tissue secretions profile towards an inflammatory one, which may have implications in adipogenesis. It has been demonstrated that members of the TNFα superfamily inhibit differentiation of SGBS preadipocytes towards adipocytes [27–29].

Adipogenic differentiation of SGBS cells has also been investigated at the transcriptomic [30, 31], proteomic [30, 32], and secretomic [32–37] level.

### Browning

Upon certain stimuli such as treatment with beta-adrenergic agonists or thiazolidinediones (TZDs), white adipocytes can acquire characteristics of brown adipocytes including expression of uncoupling protein 1 (UCP1) and mitochondrial uncoupling, in a process referred to as browning [38]. We and others [39, 40] have demonstrated that SGBS cells undergo functional browning using the standard differentiation protocol which includes the usage of rosiglitazone as inducer of PPARγ activity. Due to their high energy demand, thermogenic adipocytes are of major interest to improve energy balance and metabolism in the context of obesity [38]. Thus, identification of genes regulating the thermogenic differentiation program in human adipocytes are currently investigated. Using lentiviral-mediated stable gene knockdown in SGBS cells, we found that fat mass and obesity associated (FTO), the major obesity gene, inhibits adipocyte browning [41]. We further identified TENM2 [42] and members of the LTBP family [12] as regulators of UCP1 expression and function after comparing expression patterns of human white and brown adipocyte progenitor cells [43]. As human brown adipocytes with high UCP1 expression are hardly available, we recently generated SGBS cells overexpressing UCP1 [44]. These cells show strongly increased lipolysis-driven mitochondrial uncoupling demonstrating that SGBS cells per se provide the cellular equipment to support UCP1 activity. Furthermore, UCP1 overexpression results in increased basal glucose uptake, in line with data from murine adipocytes [44].

In comparison to differentiated primary human ASCs, SGBS cells express higher levels of the UCP1. The reason for this has not been addressed, but might reflect the young age of the SGBS cell donor. Of note, it has been demonstrated earlier that cellular senescence contributes to the low ability of ASCs from elderly donors to undergo browning [45].

### Insulin sensitivity

Peripheral insulin resistance is a hallmark of type 2 diabetes mellitus and the metabolic syndrome, leading to reduced uptake of glucose in skeletal muscle as well as adipose tissue, further resulting in elevated glucose levels. Insulin controls adipocyte metabolism on different levels including uptake of glucose, lipid synthesis and storage, free fatty acid release as well as secretion of adipokines. Thus, SGBS cells have been frequently used as a human model system to study insulin sensitivity/resistance, as the fully differentiated cells express high levels of the insulin-dependent glucose transporter GLUT4 and are sensitive to insulin in terms of glucose uptake and activation of the insulin receptor signaling pathway.

Different models of insulin resistance induction have been applied to SGBS cells including chronic high insulin and/or glucose [14, 32] or pro-inflammatory factors such as interferon gamma [46, 47], TNFα [48], or IL-29 [49]. Thereby, some key pathways could be identified which mediated these effects, e.g., the JAK/STAT pathway in IFNγ mediated insulin resistance [47]. Using SGBS cells and murine in vitro models, Caspase 1 has been previously identified as a key mediator of IL-1β-driven insulin resistance [50].

Very recently, SGBS cells were used to screen for functional effects of causal gene candidates for insulin resistance previously identified by genome-wide association studies [51]. Using Crispr/Cas9 mediated knockout, the role of 12 genes regarding adipogenesis, lipid metabolism, and insulin sensitivity was evaluated, demonstrating how SGBS cells can be used as a screening tool.

Recent data using murine as well as SGBS adipocytes demonstrate a key role of COX2-PGE2-EP3 signaling in the development of adipose tissue inflammation and insulin resistance [52]. Later, the same group showed that COX2 signaling in adipocytes is a key mediator of macrophage migration inhibitor factor (MIF) activation and promotes phenotypic switch of adipose macrophages towards an inflammatory phenotype [53].

We could recently demonstrate that miRNA-146a is involved in the regulation of adipocyte insulin sensitivity by targeting the natriuretic peptide receptor NPR3 [54].

### Adipose tissue inflammation

Obesity is associated with a chronic, low-grade inflammation in the adipose tissue [55, 56]. In this context, SGBS cells have been used to investigate aspects of the underlying pathophysiology. SGBS cells have been treated either with inflammatory cytokines or chemokines or were cultivated with immune cells to mimic a proinflammatory environment.

Globally, cultivation of SGBS cells with macrophage-conditioned medium (CM) leads to regulation of genes involved in inflammation, macrophage infiltration, lipid formation/accumulation, and glucose uptake [57]. Interestingly, several members of matrix metalloproteinases (MMP) – especially the interstitial collagenase MMP 1 and the stromelysin MMP 3 - were strongly induced and secreted by SGBS adipocytes upon CM treatment, suggesting regulation of tissue remodeling by macrophages [57]. Regulation of MMP expression upon TNFα treatment has also been found in another study [58]. In line, inhibition of *MFA5P* in SGBS results in reduced expression of inflammatory marker genes [59].

Direct co-culture of SGBS with macrophages revealed that macrophages further promote adipose tissue inflammation and insulin resistance by inducing adipocyte apoptosis via blocking Akt2 signalling [60]. Although the complete set of factors contributing to adipose tissue dysfunction has not been fully unraveled, the role of inflammatory mediators such as TNFα [48, 61–63], TRAIL [64], IFNγ [46], IL-1α [62], and LPSBP [65] have been tested in SGBS cells in regard to function and marker expression so far.

Previously, we have shown a novel mechanism in a patient with acquired autoimmune lipodystrophy [66].

Regression of adipose tissue was accompanied by lymphohistiocytic infiltration/inflammation and increased serum levels of inflammatory cytokines interferon-gamma and TNF-alpha. Using SGBS cells in in vitro studies we demonstrated that interferon-gamma and TNF-alpha are able to up-regulate CD95 expression and enhance CD95-death-inducing signaling complex formation resulting in a robust sensitization for CD95-mediated apoptosis. These results again emphasize the clinical relevance of the cell model.

Micro RNAs (miRNA) have gained substantial interest as fine-tuners of adipose tissue function within the last years. We could demonstrate that miR-146a is acting as a suppressor of inflammatory responses in SGBS adipocytes [67].

SGBS cells were also used to test the effect of different natural compounds on inflammation, including theobromine [68], olive oil polyphenols [69], grape seed procyanidins [70], and resveratrol [71].

### Adipokine secretion

In addition to its function as a storage organ, adipose tissue has important endocrine functions with impact on systemic metabolism. It is estimated that more than 600 factors are released from the adipose tissue, the so-called adipokines. Under pathologic conditions such as the metabolic syndrome, the pattern of adipokine secretion is changed dramatically towards a proinflammatory one [72].

Several studies have been conducted using SGBS cells regarding the secretion of adiponectin [73–76]. Blood levels of adiponectin are low in patients with features of the metabolic syndrome [77], and treatment with thiazolidinediones (TZDs), which had been used to improve insulin sensitivity, result in increased adiponectin levels [78]. Using different in vitro models including SGBS cells, it has been demonstrated that treatment with pioglitazone strongly induces the formation and secretion of high-molecular weight (HMW) adiponectin from adipocytes [73]. The same group later found that calcium is a critical mediator of HMW adiponectin formation [74]. Efficient secretion of HMW adiponectin is dependent on post-translational modification [79]. An Australian study could recently identify collagen beta (1-O) galactosyltransferase 1 (GLT25D1) as a key enzyme in the secretion process of HWM adiponectin from SGBS cells [76]. The secretion of other adipokines such as ANGPTL4 [80], RBP4 [81], apolipoprotein E [82], Zinc-α 2-glycoprotein [83], and IL-33 [84] were part of recent studies using SGBS cells as model system. Very recently, a MRM-based multiplexed quantification assay was used to identify further adipokines and apolipoproteins secreted from SGBS cells [85].

### Co-culture models/cell-cell communication

Co-culture models are helpful tools to understand cell-cell-communication in various diseases. Obesity is characterized by chronic low-grade inflammation paralleled by infiltration of pro-inflammatory macrophages into the adipose tissue. This inflammation is the key event in the development of obesity-associated disorders, such as insulin resistance. In vitro co-culture of adipocytes and macrophages have been frequently applied in this context (as reviewed in [86]). The co-culture of SGBS cells with human THP-1 in vitro differentiated monocytes or with conditioned media made from these cells, provide a homogenous, highly reproducible human system for the study of adipose inflammation in vitro [60, 87]. With this, we could identify a novel mechanism of macrophage-adipocyte interaction beneath triggering insulin resistance, i.e., the macrophage mediated induction of adipocyte apoptosis and subsequent phagocytosis of adipocytes by macrophages [60, 86]. This in vitro model reflects the in vivo situation, as in the adipose tissue dying adipocytes have been shown to be surrounded by macrophages [60].

Further, macrophages completely inhibit adipogenic differentiation of SGBS cells [86], induce an inflammatory secretion pattern in SGBS adipocytes [86], and modulate adipocyte metabolism in dependence of the macrophage phenotype [87]. This model has been also expanded to co-cultures with primary macrophages [88].

The cross-talk of preadipocytes and adipocytes with hepatocytes has been addressed by treating HepG2 cells with supernatants from SGBS cells [89]. Interestingly, it was shown that IL-1β present in conditioned media from SGBS cells trigger the expression of PAI-1 and fibrinogen in hepatocytes, potentially promoting secretion of hepatic coagulation factors [89].

Co-culture of SGBS cells with MCF-7 breast cancer cells was performed to study the role of obesity in breast cancer development [90]. While this induced hypoxia-related genes such as HIF1α in SGBS cells, co-culture induces genes of endothelial-to-mesenchymal transition in MCF-7 cells, supporting an important role of obesity in driving breast cancer aggressiveness [90].

In another study, secretion of adipokines has been studied in response to co-culture with or conditioned media of intestinal epithelial cells [91]. Interestingly, co-culture of SGBS cells with Caco-2 cells or conditioned media induced expression and secretion of leptin as well as adiponectin, suggesting that humoral factors from enterocytes affect the adipocyte secretion profile.

### Limitations of the cell model

The SGBS cell model has its limitations in some aspects. First, neither the genetic variants associated with the phenotype of the patient SGBS cells are derived from nor any potential somatic genetic variants(s) leading to the characteristics of the SGBS cells in vivo have been resolved yet, so further investigations addressing this are warranted. Second, SGBS cells have been isolated from the subcutaneous adipose tissue, which should be considered when comparing these cells with cells isolated from other adipose tissue depots. Further, adipogenic differentiation of SGBS cells is almost completely dependent on a strong PPARγ agonist, e.g., rosiglitazone. This probably might be due to their prolonged expansion in cell culture. Beneath induction of adipogenesis, rosiglitazone induces expression of UCP1 in adipocytes reminiscent of a beige adipocyte phenotype [8]. Although rosiglitazone is used in many differentiation protocols, this should be kept in mind when using this cell model, and validation of key experiments in additional models may be advantageous.

## Conclusions and outlook

The systematic characterization of the human SGBS cell model confirms that this cell strain is a versatile in vitro model to study all aspects of human adipocyte biology. According to the literature reviewed herein, SGBS cells are successfully used in different fields of adipocyte biology research. The strain provides an almost unlimited source of preadipocytes with high proliferative and differentiation capacity. SGBS cells are suitable for culture in 96-well and 384-well plates, enabling high-throughput analysis for large-scale drug testing. SGBS cells show optimal behavior in key metabolic assays under chemically-defined conditions, comparable to primary human adipocytes. We also speculate that SGBS cells may be used in the future to generate 3D cell culture models of adipose tissue or in combination with keratinocytes, fibroblast and endothelial cells to generate a human skin model.

## Materials and methods relevant for Part A

### Cell culture and adipogenic differentiation

SGBS cells were cultured and differentiated into adipocytes using a protocol described before [1] with modifications. Cells were seeded into cell culture vessels in DMEM:F12 (Thermofisher, Waltham, USA) containing 33 µM biotin, 17 µM panthotenate, and 10% FCS, until reaching subconfluence. To induce adipogenic differentiation, cells were washed once with three volumes of PBS und cultured thereafter in serum-free DMEM:F12 medium supplemented with 10 μg/ml apo-transferrin, 10 nM insulin, 200 pM T3, and 1 μM cortisol. For the first 4 days, 2 μM rosiglitazone, 250 μM isobutylmethylxanthine, and 25 nM dexamethasone were added. Medium was replaced thereafter every 2–3 days.

### Isolation of adipose stromal cells from children

Human adipose stromal cells were isolated from *n* = 4 infants undergoing herniotomy. The study was approved by the ethical committee of Ulm University and all patient’s caregivers provided written informed consent. Subcutaneous adipose tissue biopsied (approx. 50–200 mg) were minced into fine pieces and digested with 200 U/ml collagenase (Type I, Merck, Darmstadt, Germany) in DMEM:F12 for 60 min at 37 °C. Residual tissue was removed by filtering through a 200 µm strainer, and stromal cells were separated from mature adipocytes by centrifugation (200 g, 10 min). After removal of erythrocytes by hypotonic lysis, cells were pelleted again and resuspended in growth medium (DMEM:Hams F12 (1:1) supplemented with antibiotics, 10% fetal calf serum, 33 µM biotin and 17 µM panthotenate. Plastic-adherent cells were propagated in growth medium containing 2.5 ng/l basic fibroblast growth factor (FGF2) for two passages and then used for experiments.

### Stainings

SGBS cells were seeded on coverslips (Falcon, Thermofisher), and differentiated into adipocytes. On different days of differentiation, cells were washed with PBS and fixed with 4% formaldehyde/PBS for 10 min at room temperature. For OilRed O (ORO) stainings, cells were washed with 60% 2-propanol and stained with ORO solution (2 g/l in 60% 2-propanol) for 10 min and washed with water. For BODIPY stainings, cells were incubated with BODIPY 493/503 (Thermofisher) and Hoechst33342 (Thermofisher) in PBS for 10 minutes and washed with PBS afterwards. Specimens were observed using a Keyence BZ6000 microscope (Keyence, Osaka, Japan).

### Glucose metabolism

In vitro differentiated adipocytes were washed with PBS and incubated in serum-free DMEM:F12 overnight. On the day of measurement, medium was replaced with glucose-free Krebs-Ringer buffer (130 mM NaCl, 10 mM MgSO_4_, 2.5 mM NaH_2_PO_4_, 4.6 mM KCl, 2.5 mM CaCl_2_, 2.5 mM sodium pyruvate, 5 mM HEPES, pH 7.4). To determine insulin-dependent glucose uptake, cells were treated with increasing concentrations of human recombinant insulin (Thermofisher) for 15 min. Subsequently, ^14^C-2-deoxy-D-glucose (0.2 μCi/well, PerkinElmer, Waltham, USA) was added and the cells were incubated for 15 min at 37 °C. Subsequently, cells were washed with ice-cold PBS and harvested in 100 mM NaOH. Incorporation of ^14^C-2-deoxy-D-glucose was measured on a β-counter.

To study glucose incorporation into cellular lipids, adipocytes were incubated ^14^C-D-Glucose for 24 hours. Subsequently, cells were washed with ice-cold PBS and harvested in 100 mM NaOH. Incorporation of [14 C]-2-deoxy-D-glucose was measured on a β-counter.

### Expression analysis

RNA was isolated using the Direct-Zol RNA Kit (Zymo Research, Irvine, USA). 0.5 μg of total RNA was reverse transcribed using SuperScript II Reverse Transcriptase (Thermofisher). Relative expression of target genes was analyzed by quantitative real-time PCR using the ssoAdvanced Universal SYBR Green Supermix on a CFX Connect Real Time PCR Detection System (BioRad, Munich, Germany). Expression values were calculated using the dCt method with hypoxanthine-guanine phosphoribosyltransferase (HPRT) as a reference gene.

Total protein was extracted by washing with ice-cold PBS and scraping the cells in a lysis buffer (10 mM Tris-HCl at pH 7.5, 150 mM sodium chloride, 2 mM EDTA, 1% TX-100, 10% glycerol, 1X cOmplete Protease Inhibitors (Roche, Mannheim, Germany)). After 30 min incubation on ice, cell debris were pelleted by centrifugation. Protein content in the supernatants was determined using the Bradford Protein Assay (BioRad). For immunodetection, 10–20 μg of protein were separated by SDS-PAGE on Bolt Bis-Tris Plus Gels (Thermofisher) and transferred to nitrocellulose membranes by Western Blotting (Thermofisher). The following antibodies were used: mouse anti-UCP1 (R&D Systems #536435, Minneapolis, UCA), rabbit anti-leptin (BioVendor #RD181001220), rabbit anti-perilipin A (Abcam #ab3526), rabbit anti-PPARG (Cell Signaling Technology #2443), rabbit anti-adiponectin (GeneTex #GTX112777), hFAB rhodamine anti-Tubulin (BioRad #12004165).

### Cellular flux analysis

Cells were plated in 96-well cell culture microplates (XFe96, Agilent Technologies, Santa Clara, USA) and differentiated into adipocytes. On the day of measurement (days 0 and 14), the cells were incubated for 1 h in bicarbonate-free DMEM medium containing 5 mM HEPES, 10 mM glucose, 1 mM pyruvate, 2 mM glutamine. Oxygen consumption and extracellular acidification rates (OCR and ECAR) were measured simultaneously using a Seahorse XFe96 Flux Analyzer (Agilent Technologies). Uncoupled (proton leak) respiration was profiled by injecting 2 μM oligomycin (inhibiting the ATP synthase), and full substrate oxidation capacity was determined by injecting 4 μM carbonylcyanide-p-trifluoromethoxyphenylhydrazone (FCCP, a chemical uncoupler). Non-mitochondrial respiration was determined by injecting 1.5 μM antimycin A and 1.5 μM rotenone (inhibiting electron flux through complex I and III). Full glycolytic capacity was determined by injecting antimycin A, rotenone and 20 µM monensin. OCR and ECAR were determined by machine algorithms and plotted against time. Data were normalized for DNA content using picoGreen staining (Thermofisher). ATP production rates were calculated from OCR and ECAR rates assuming a P/O ratio of 2.75.

## Supplementary information

Supplementary table 1

## Data Availability

Data are available within the article or its supplementary materials. Details of primer sequences and antibodies used in this study are available from the authors.
