# Supplementary material for: 20 Years with SGBS cells - a versatile in vitro model of human adipocyte biology
Source: Int J Obes (Lond). 2022 Aug 19;46(11):1939–47. doi: 10.1038/s41366-022-01199-9 (PMC9584814; doi:10.1038/s41366-022-01199-9)
Supplement: Supplementary file 1 — Supplementary table 1 [file 41366_2022_1199_MOESM1_ESM.docx]

Supplemental Material for

Tews D., et al.: “20 Years with SGBS cells - a versatile *in vitro* model of human adipocyte biology”

**Supplemental Table 1:** Complete list of original articles publishing data obtained with SGBS cells.

1 Ahonen MA, Haridas PAN, Mysore R, Wabitsch M, Fischer-Posovszky P, Olkkonen VM. miR-107 inhibits CDK6 expression, differentiation, and lipid storage in human adipocytes. Mol Cell Endocrinol. 2019 Jan;479:110–6.

2 Aiwei YB, Behjatolah MK, Hedges RA, Rogers LJ, Kadlubar SA, Thomas KE. Adipocyte hypoxia promotes epithelial-mesenchymal transition-related gene expression and estrogen receptor-negative phenotype in breast cancer cells. Oncol Rep. 2015 Jun;33(6):2689–94.

3 Alex S, Lange K, Amolo T, Grinstead JS, Haakonsson AK, Szalowska E, et al. Short-Chain Fatty Acids Stimulate Angiopoietin-Like 4 Synthesis in Human Colon Adenocarcinoma Cells by Activating Peroxisome Proliferator-Activated Receptor  . Mol Cell Biol. 2013 Apr;33(7):1303–16.

4 Allott EH, Oliver E, Lysaght J, Gray SG, Reynolds J V., Roche HM, et al. The SGBS cell strain as a model for the in vitro study of obesity and cancer. Clin Transl Oncol. 2012 Oct;14(10):774–82.

5 Alvarez MS, Fernandez-Alvarez A, Cucarella C, Casado M. Stable SREBP-1a knockdown decreases the cell proliferation rate in human preadipocyte cells without inducing senescence. Biochem Biophys Res Commun. 2014 Apr;447(1):51–6.

6 Arianti R, Vinnai BÁ, Tóth BB, Shaw A, Csősz É, Vámos A, et al. ASC-1 transporter-dependent amino acid uptake is required for the efficient thermogenic response of human adipocytes to adrenergic stimulation. FEBS Lett. 2021 Aug;595(16):2085–98.

7 Ballak DB, van Asseldonk EJP, van Diepen JA, Jansen H, Hijmans A, Joosten LAB, et al. TLR-3 is Present in Human Adipocytes, but Its Signalling is Not Required for Obesity-Induced Inflammation in Adipose Tissue In Vivo. PLoS One. 2015 Apr;10(4):e0123152.

8 Banga A, Bodles AM, Rasouli N, Ranganathan G, Kern PA, Owens RJ. Calcium is involved in formation of high molecular weight adiponectin. Metab Syndr Relat Disord. 2008 Jun;6(2):103–11.

9 Bao Y, Bing C, Hunter L, Jenkins JR, Wabitsch M, Trayhurn P. Zinc-α 2-glycoprotein, a lipid mobilizing factor, is expressed and secreted by human (SGBS) adipocytes. FEBS Lett. 2005 Jan;579(1):41–7.

10 Baraban E, Chavakis T, Hamilton BS, Sales S, Wabitsch M, Bornstein SR, et al. Anti-inflammatory properties of bone morphogenetic protein 4 in human adipocytes. Int J Obes. 2016 Feb;40(2):319–27.

11 Berger S, Ceccarini G, Scabia G, Barone I, Pelosini C, Ferrari F, et al. Lipodystrophy and obesity are associated with decreased number of T cells with regulatory function and pro-inflammatory macrophage phenotype. Int J Obes. 2017 Nov;41(11):1676–84.

12 Bernhard F, Landgraf K, Klöting N, Berthold a., Büttner P, Friebe D, et al. Functional relevance of genes implicated by obesity genome-wide association study signals for human adipocyte biology. Diabetologia. 2013 Feb;56(2):311–22.

13 Bodles AM, Banga A, Rasouli N, Ono F, Kern PA, Owens RJ. Pioglitazone increases secretion of high-molecular-weight adiponectin from adipocytes. Am J Physiol - Endocrinol Metab. 2006;291(5). DOI: 10.1152/ajpendo.00187.2006

14 Bour S, Daviaud D, Gres S, Lefort C, Prévot D, Zorzano A, et al. Adipogenesis-related increase of semicarbazide-sensitive amine oxidase and monoamine oxidase in human adipocytes. Biochimie. 2007 Aug;89(8):916–25.

15 Breitling C, Gross A, Böttner P, Weise S, Schleinitz D, Kiess W, et al. Genetic contribution of variants near SORT1 and APOE on LDL cholesterol independent of obesity in children. PLoS One. 2015 Sep;10(9). DOI: 10.1371/journal.pone.0138064

16 Britton LJ, Bridle K, Jaskowski LA, He J, Ng C, Ruelcke JE, et al. Iron Inhibits the Secretion of Apolipoprotein E in Cultured Human Adipocytes. CMGH. 2018 Jan;6(2):215-217.e8.

17 Cannon ME, Currin KW, Young KL, Perrin HJ, Vadlamudi S, Safi A, et al. Open chromatin profiling in adipose tissue marks genomic regions with functional roles in cardiometabolic traits. G3 Genes, Genomes, Genet. 2019 Aug;9(8):2521–33.

18 Cannon ME, Duan Q, Wu Y, Zeynalzadeh M, Xu Z, Kangas AJ, et al. Trans-ancestry fine mapping and molecular assays identify regulatory variants at the ANGPTL8 HDL-C GWAS locus. G3 Genes, Genomes, Genet. 2017 Sep;7(9):3217–27.

19 Carayol J, Chabert C, Di Cara A, Armenise C, Lefebvre G, Langin D, et al. Protein quantitative trait locus study in obesity during weight-loss identifies a leptin regulator. Nat Commun. 2017 Dec;8(1). DOI: 10.1038/s41467-017-02182-z

20 Carpi S, Scoditti E, Massaro M, Polini B, Manera C, Digiacomo M, et al. The extra-virgin olive oil polyphenols oleocanthal and oleacein counteract inflammation-related gene and mirna expression in adipocytes by attenuating nf-κb activation. Nutrients. 2019 Dec;11(12). DOI: 10.3390/nu11122855

21 Casadei L, Calore F, Braggio DA, Zewdu A, Deshmukh AA, Fadda P, et al. MDM2 derived from dedifferentiated liposarcoma extracellular vesicles induces MMP2 production from preadipocytes. Cancer Res. 2019;79(19):4911–22.

22 Chacón MR, Ceperuelo-Mallafré V, Maymó-Masip E, Mateo-Sanz JM, Arola L, Guitiérrez C, et al. Grape-seed procyanidins modulate inflammation on human differentiated adipocytes in vitro. Cytokine. 2009 Aug;47(2):137–42.

23 Chan PC, Hsiao FC, Chang HM, Wabitsch M, Shiuan Hsieh P. Importance of adipocyte cyclooxygenase-2 and prostaglandin E2-prostaglandin E receptor 3 signaling in the development of obesity-induced adipose tissue inflammation and insulin resistance. FASEB J. 2016 Jun;30(6):2282–97.

24 Chan PC, Wu TN, Chen YC, Lu CH, Wabitsch M, Tian YF, et al. Targeted inhibition of CD74 attenuates adipose COX-2-MIF-mediated M1 macrophage polarization and retards obesity-related adipose tissue inflammation and insulin resistance. Clin Sci. 2018 Jul;132(14):1581–96.

25 Chen Z, Yu H, Shi X, Warren CR, Lotta LA, Friesen M, et al. Functional Screening of Candidate Causal Genes for Insulin Resistance in Human Preadipocytes and Adipocytes. Circ Res. 2020;330–46.

26 Cyr Y, Lamantia V, Bissonnette S, Burnette M, Besse-Patin A, Demers A, et al. Lower plasma PCSK9 in normocholesterolemic subjects is associated with upregulated adipose tissue surface-expression of LDLR and CD36 and NLRP3 inflammasome. Physiol Rep. 2021 Feb;9(3). DOI: 10.14814/phy2.14721

27 Dahlhaus M, Roos J, Engel D, Tews D, Halbgebauer D, Funcke J-B, et al. CD90 Is Dispensable for White and Beige/Brown Adipocyte Differentiation. Int J Mol Sci. 2020 Oct;21(21):7907.

28 Davis JP, Vadlamudi S, Roman TS, Zeynalzadeh M, Iyengar AK, Mohlke KL. Enhancer deletion and allelic effects define a regulatory molecular mechanism at the VLDLR cholesterol GWAS locus. Hum Mol Genet. 2019;28(6):888–95.

29 Davoodi-Semiromi A, Hassanzadeh A, Wasserfall CH, Droney A, Atkinson M. Tyrphostin AG490 agent modestly but significantly prevents onset of type 1 in NOD mouse; Implication of immunologic and metabolic effects of a jak-stat pathway inhibitor. J Clin Immunol. 2012 Oct;32(5):1038–47.

30 De Heredia FP, Stuart Wood I, Trayhurn P. Hypoxia stimulates lactate release and modulates monocarboxylate transporter (MCT1, MCT2, and MCT4) expression in human adipocytes. Pflugers Arch Eur J Physiol. 2010 Feb;459(3):509–18.

31 Derdemezis CS, Kiortsis DN, Tsimihodimos V, Petraki MP, Vezyraki P, Elisaf MS, et al. Effect of Plant Polyphenols on Adipokine Secretion from Human SGBS Adipocytes. Biochem Res Int. 2011;2011:1–5.

32 Díaz-Delfín J, Domingo P, Wabitsch M, Giralt M, Villarroya F. HIV-1 Tat protein impairs adipogenesis and induces the expression and secretion of proinflammatory cytokines in human SGBS adipocytes. Antivir Ther. 2012;17(3):529–40.

33 Dijk W, Mattijssen F, De La Rosa Rodriguez M, Valdes AL, Loft A, Mandrup S, et al. Hypoxia-inducible lipid droplet-associated is not a direct physiological regulator of lipolysis in adipose tissue. Endocrinology. 2017 May;158(5):1231–51.

34 DiStefano MT, Roth Flach RJ, Senol-Cosar O, Danai L V., Virbasius J V., Nicoloro SM, et al. Adipocyte-specific Hypoxia-inducible gene 2 promotes fat deposition and diet-induced insulin resistance. Mol Metab. 2016 Dec;5(12):1149–61.

35 Do MS, Jeong HS, Choi BH, Hunter L, Langley S, Pazmany L, et al. Inflammatory gene expression patterns revealed by DNA microarray analysis in TNF-α-treated SGBS human adipocytes. Yonsei Med J. 2006 Oct;47(5):729–36.

36 Doan-Xuan QM, Sarvari AK, Fischer-Posovszky P, Wabitsch M, Balajthy Z, Fesus L, et al. High content analysis of differentiation and cell death in human adipocytes. Cytom Part A. 2013 Oct;83(10):933–43.

37 Dong SS, Yao S, Chen YX, Guo Y, Zhang YJ, Niu HM, et al. Detecting epistasis within chromatin regulatory circuitry reveals CAND2 as a novel susceptibility gene for obesity. Int J Obes. 2019 Mar;43(3):450–6.

38 Donkers JM, Kooijman S, Slijepcevic D, Kunst RF, Roscam Abbing RLP, Haazen L, et al. NTCP deficiency in mice protects against obesity and hepatosteatosis. JCI Insight. 2019 Jul;4(14). DOI: 10.1172/jci.insight.127197

39 Dünner N, Quezada C, Berndt FA, Cánovas J, Rojas C V. Angiotensin II Signaling in Human Preadipose Cells: Participation of ERK1,2-Dependent Modulation of Akt. PLoS One. 2013 Oct;8(10). DOI: 10.1371/journal.pone.0075440

40 Enlund E, Fischer S, Handrick R, Otte K, Debatin KM, Wabitsch M, et al. Establishment of lipofection for studying miRNA function in human adipocytes. PLoS One. 2014 May;9(5). DOI: 10.1371/journal.pone.0098023

41 Erman A, Wabitsch M, Goodyer CG. Human growth hormone receptor (GHR) expression in obesity: II. Regulation of the human GHR gene by obesity-related factors. Int J Obes. 2011 Dec;35(12):1520–9.

42 Ernst J, Gert K, Kraus FB, Rolle-Kampczyk UE, Wabitsch M, Dehghani F, et al. Androstenedione changes steroidogenic activity of SGBS cells. Endocr Connect. 2020 Jun DOI: 10.1530/ec-19-0549

43 Ernst J, Grabiec U, Falk K, Dehghani F, Schaedlich K. The endocrine disruptor DEHP and the ECS: Analysis of a possible crosstalk. Endocr Connect. 2020;9(2):101–10.

44 Esteves CL, Kelly V, Breton A, Taylor AI, West CC, Donadeu FX, et al. Proinflammatory cytokine induction of 11β-hydroxysteroid dehydrogenase type 1 (11β-HSD1) in human adipocytes is mediated by MEK, C/EBPβ, and NF-κB/RelA. J Clin Endocrinol Metab. 2014 Jan;99(1). DOI: 10.1210/jc.2013-1708

45 Faber DR, Kalkhoven E, Westerink J, Bouwman JJ, Monajemi HM, Visseren FLJ. Conditioned media from (pre)adipocytes stimulate fibrinogen and PAI-1 production by HepG2 hepatoma cells. Nutr Diabetes. 2012 Dec;2(DECEMBER). DOI: 10.1038/nutd.2012.25

46 Fathzadeh M, Li J, Rao A, Cook N, Chennamsetty I, Seldin M, et al. FAM13A affects body fat distribution and adipocyte function. Nat Commun. 2020 Dec;11(1). DOI: 10.1038/s41467-020-15291-z

47 Felicidade I, Sartori D, Coort SLM, Semprebon SC, Niwa AM, D’Epiro GFR, et al. Role of 1α,25-Dihydroxyvitamin D 3 in Adipogenesis of SGBS Cells: New Insights into Human Preadipocyte Proliferation. Cell Physiol Biochem. 2018 Aug;48(1):407–18.

48 Finlin BS, Bodles-Brakhop AM, Yao-Borengasser A, Zhu B, Starnes CP, McGehee RE, et al. Regulation of small ubiquitin-like modifier-1, nuclear receptor coreceptor, histone deacetylase 3, and peroxisome proliferator-activated receptor-γ in human adipose tissue. Metab Syndr Relat Disord. 2012 Aug;10(4):312–7.

49 Fischer-Posovszky P, Kukulus V, Zulet MA, Debatin KM, Wabitsch M. Conjugated linoleic acids promote human fat cell apoptosis. Horm Metab Res. 2007 Mar;39(3):186–91.

50 Fischer-Posovszky P, Newell FS, Wabitsch M, Tornqvist HE. Human {SGBS} Cells - a Unique Tool for Studies of Human Fat Cell Biology. Obes Facts Eur J Obes. 2008 Sep;1(4):184–9.

51 Fischer-Posovszky P, Tews D, Horenburg S, Debatin K-MM, Wabitsch M. Differential function of Akt1 and Akt2 in human adipocytes. Mol Cell Endocrinol. 2012 Jul;358(1):135–43.

52 Friebe D, Neef M, Erbs S, Dittrich K, Kratzsch J, Kovacs P, et al. Retinol binding protein 4 (RBP4) is primarily associated with adipose tissue mass in children. Int J Pediatr Obes. 2011 Jun;6(2–2). DOI: 10.3109/17477166.2010.491228

53 Friebe D, Löffler D, Schönberg M, Bernhard F, Büttner P, Landgraf K, et al. Impact of metabolic regulators on the expression of the obesity associated genes FTO and NAMPT in human preadipocytes and adipocytes. PLoS One. 2011;6(6):1–6.

54 Friesen M, Warren CR, Yu H, Toyohara T, Ding Q, Florido MHC, et al. Mitoregulin Controls β-Oxidation in Human and Mouse Adipocytes. Stem Cell Reports. 2020 Apr;14(4):590–602.

55 Fuggetta MP, Zonfrillo M, Villivà C, Bonmassar E, Ravagnan G. Inflammatory microenvironment and adipogenic differentiation in obesity: The inhibitory effect of theobromine in a model of human obesity in vitro. Mediators Inflamm. 2019;2019. DOI: 10.1155/2019/1515621

56 Funcke JB, Zoller V, El Hay MA, Debatin KM, Wabitsch M, Fischer-Posovszky P. TNF-related apoptosis-inducing ligand promotes human preadipocyte proliferation via ERK1/2 activation. FASEB J. 2015 Jul;29(7):3065–75.

57 Galhardo M, Sinkkonen L, Berninger P, Lin J, Sauter T, Heinäniemi M. ChIP-seq profiling of the active chromatin marker H3K4me3 and PPARγ, CEBPα and LXR target genes in human SGBS adipocytes. Genomics Data. 2014;2:230–6.

58 Galhardo M, Sinkkonen L, Berninger P, Lin J, Sauter T, Heinäniemi M. Integrated analysis of transcript-level regulation of metabolism reveals disease-relevant nodes of the human metabolic network. Nucleic Acids Res. 2014 Feb;42(3):1474–96.

59 Galhardo M, Sinkkonen L, Berninger P, Lin J, Sauter T, Heinäniemi M. Transcriptomics profiling of human SGBS adipogenesis. Genomics Data. 2014;2:246–8.

60 García-Beltran C, Cereijo R, Quesada-López T, Malpique R, López-Bermejo A, De Zegher F, et al. Reduced circulating levels of chemokine CXCL14 in adolescent girls with polycystic ovary syndrome: Normalization after insulin sensitization. BMJ Open Diabetes Res Care. 2020 Feb;8(1). DOI: 10.1136/bmjdrc-2019-001035

61 Gavaldà-Navarro A, Domingo P, Viñas O, Mampel T. Expression of human and mouse adenine nucleotide translocase (ANT) isoform genes in adipogenesis. Int J Biochem Cell Biol. 2015 Jul;64:34–44.

62 Gavaldà-Navarro A, Mampel T, Viñas O. Changes in the expression of the human adenine nucleotide translocase isoforms condition cellular metabolic/proliferative status. Open Biol. 2016 Feb;6(2). DOI: 10.1098/rsob.150108

63 Geiger K, Muendlein A, Stark N, Saely CH, Wabitsch M, Fraunberger P, et al. Hypoxia induces apelin expression in human adipocytes. Horm Metab Res. 2011 Jun;43(6):380–5.

64 Geiger K, Leiherer A, Muendlein A, Stark N, Geller-Rhomberg S, Saely CH, et al. Identification of Hypoxia-Induced Genes in Human SGBS Adipocytes by Microarray Analysis. PLoS One. 2011 Oct;6(10):e26465.

65 Giralt A, Hondares E, Villena JA, Ribas F, Díaz-Delfín J, Giralt M, et al. Peroxisome proliferator-activated receptor-γ coactivator-1α controls transcription of the Sirt3 gene, an essential component of the thermogenic brown adipocyte phenotype. J Biol Chem. 2011 May;286(19):16958–66.

66 Gottmann P, Ouni M, Saussenthaler S, Roos J, Stirm L, Jähnert M, et al. A computational biology approach of a genome-wide screen connected miRNAs to obesity and type 2 diabetes. Mol Metab. 2018 May;11:145–59.

67 Greither T, Wenzel C, Jansen J, Kraus M, Wabitsch M, Behre HM. MiR-130a in the adipogenesis of human SGBS preadipocytes and its susceptibility to androgen regulation. Adipocyte. 2020 Jan;9(1):197–205.

68 Grigem S, Fischer-Posovszky P, Debatin KM, Loizon E, Vidal H, Wabitsch M. The effect of the {HIV} protease inhibitor ritonavir on proliferation, differentiation, lipogenesis, gene expression and apoptosis of human preadipocytes and adipocytes. Horm Metab Res. 2005 Oct;37(10):602–9.

69 Guennoun A, Kazantzis M, Thomas R, Wabitsch M, Tews D, Seetharama Sastry K, et al. Comprehensive molecular characterization of human adipocytes reveals a transient brown phenotype. J Transl Med. 2015 Dec;13(1):135.

70 Haider N, Dusseault J, Rudich A, Larose L. Nck2, an unexpected regulator of adipogenesis. Adipocyte. 2017 Apr;6(2):154–60.

71 Halbgebauer D, Roos J, Funcke JB, Neubauer H, Hamilton BS, Simon E, et al. Latent TGFβ-binding proteins regulate UCP1 expression and function via TGFβ2. Mol Metab. 2021;53(September):101336.

72 Halbgebauer D, Dahlhaus M, Wabitsch M, Fischer-Posovszky P, Tews D. Browning capabilities of human primary adipose-derived stromal cells compared to SGBS cells. Sci Rep. 2020 Dec;10(1):9632.

73 Han CY, Tang C, Guevara ME, Wei H, Wietecha T, Shao B, et al. Serum amyloid A impairs the antiinflammatory properties of HDL. J Clin Invest. 2016 Jan;126(1):266–81.

74 Hessvik NP, Bakke SS, Smith R, Ravna AW, Sylte I, Rustan AC, et al. The liver X receptor modulator 22(S)-hydroxycholesterol exerts cell-type specific effects on lipid and glucose metabolism. J Steroid Biochem Mol Biol. 2012 Feb;128(3–5):154–64.

75 Hjorth M, Pourteymour S, Görgens SW, Langleite TM, Lee S, Holen T, et al. Myostatin in relation to physical activity and dysglycaemia and its effect on energy metabolism in human skeletal muscle cells. Acta Physiol. 2016 May;217(1):45–60.

76 Hoggard N, Cruickshank M, Moar KM, Bashir S, Mayer CD. Using gene expression to predict differences in the secretome of human omental vs. subcutaneous adipose tissue. Obesity. 2012 Jun;20(6):1158–67.

77 Horenburg S, Fischer-Posovszky P, Debatin KM, Wabitsch M. Influence of sex hormones on adiponectin expression in human adipocytes. Horm Metab Res. 2008 Nov;40(11):779–86.

78 Hossain MG, Iwata T, Mizusawa N, Shima SWN, Okutsu T, Ishimoto K, et al. Compressive force inhibits adipogenesis through COX-2-mediated down-regulation of PPARγ2 and C/EBPα. J Biosci Bioeng. 2010 Mar;109(3):297–303.

79 Huebner L, Engeli S, Wrann CD, Goudeva L, Laue T, Kielstein H. Human NK Cell Subset Functions Are Differentially Affected by Adipokines. PLoS One. 2013 Sep;8(9). DOI: 10.1371/journal.pone.0075703

80 Ishihara R, Mizuno Y, Miwa A, Hamada A, Tsuruta T, Wabitsch M, et al. Intestinal epithelial cells promote secretion of leptin and adiponectin in adipocytes. Biochem Biophys Res Commun. 2015 Mar;458(2):362–8.

81 Ishimoto K, Iwata T, Taniguchi H, Mizusawa N, Tanaka E, Yoshimoto K. D-Dopachrome tautomerase promotes IL-6 expression and inhibits adipogenesis in preadipocytes. Cytokine. 2012 Dec;60(3):772–7.

82 Iwata T, Kuribayashi K, Nakasono M, Saito-Tarashima N, Minakawa N, Mizusawa N, et al. The AMPK/mTOR pathway is involved in D-dopachrome tautomerase gene transcription in adipocytes differentiated from SGBS cells, a human preadipocyte cell line. Cytokine. 2017 Aug;96:195–202.

83 Iwata T, Taniguchi H, Kuwajima M, Taniguchi T, Okuda Y, Sukeno A, et al. The action of D-dopachrome tautomerase as an adipokine in adipocyte lipid metabolism. PLoS One. 2012 Mar;7(3). DOI: 10.1371/journal.pone.0033402

84 Jasinski-Bergner S, Büttner M, Quandt D, Seliger B, Kielstein H. Adiponectin and Its Receptors Are Differentially Expressed in Human Tissues and Cell Lines of Distinct Origin. Obes Facts. 2018 Feb;10(6):569–83.

85 Jeninga EH, Bugge A, Nielsen R, Kersten S, Hamers N, Dani C, et al. Peroxisome proliferator-activated receptor γ regulates expression of the anti-lipolytic G-protein-coupled receptor 81 (GPR81/Gpr81). J Biol Chem. 2009 Sep;284(39):26385–93.

86 Kalkhof S, Krieg L, Büttner P, Wabitsch M, Küntzel C, Friebe D, et al. In Depth Quantitative Proteomic and Transcriptomic Characterization of Human Adipocyte Differentiation Using the SGBS Cell Line. Proteomics. 2020 May;1900405.

87 Kässner F, Kirstein A, Händel N, Schmid GL, Landgraf K, Berthold A, et al. A new human adipocyte model with PTEN haploinsufficiency. Adipocyte. 2020 Jan;9(1):290–301.

88 Keuper M, Wernstedt Asterholm I, Scherer PE, Westhoff MA, Möller P, Debatin KM, et al. TRAIL (TNF-related apoptosis-inducing ligand) regulates adipocyte metabolism by caspase-mediated cleavage of PPARgamma. Cell Death Dis. 2013;4. DOI: 10.1038/cddis.2012.212

89 Keuper M, Berti L, Raedle B, Sachs S, Böhm A, Fritsche L, et al. Preadipocytes of obese humans display gender-specific bioenergetic responses to glucose and insulin. Mol Metab. 2019 Feb;20:28–37.

90 Keuper M, Blüher M, Schön MR, Möller P, Dzyakanchuk A, Amrein K, et al. An inflammatory micro-environment promotes human adipocyte apoptosis. Mol Cell Endocrinol. 2011 Jun;339(1–2):105–13.

91 Keuper M, Dzyakanchuk A, Amrein KE, Wabitsch M, Fischer-Posovszky P. THP-1 macrophages and SGBS adipocytes - a new human in vitro model system of inflamed adipose tissue. Front Endocrinol (Lausanne). 2011;2(DEC). DOI: 10.3389/fendo.2011.00089

92 Keuper M, Sachs S, Walheim E, Berti L, Raedle B, Tews D, et al. Activated macrophages control human adipocyte mitochondrial bioenergetics via secreted factors. Mol Metab. 2017 Oct;6(10):1226–39.

93 Klusóczki Á, Veréb Z, Vámos A, Fischer-Posovszky P, Wabitsch M, Bacso Z, et al. Differentiating SGBS adipocytes respond to PPARγ stimulation, irisin and BMP7 by functional browning and beige characteristics. Sci Rep. 2019 Dec;9(1). DOI: 10.1038/s41598-019-42256-0

94 Koenen TB, Stienstra R, Van Tits LJ, De Graaf J, Stalenhoef AFH, Joosten LAB, et al. Hyperglycemia activates caspase-1 and TXNIP-mediated IL-1β transcription in human adipose tissue. Diabetes. 2011 Feb;60(2):517–24.

95 Koenig C, Fischer-Posovszky P, Rojewski MT, Tews D, Schrezenmeier H, Wabitsch M, et al. Absence of CC chemokine receptors 2a and 2b from human adipose lineage cells. Mol Cell Endocrinol. 2013 Apr;369(1–2):72–85.

96 Kotnik P, Keuper M, Wabitsch M, Fischer-Posovszky P. Interleukin-1β Downregulates RBP4 Secretion in Human Adipocytes. PLoS One. 2013 Feb;8(2). DOI: 10.1371/journal.pone.0057796

97 Kraus M, Greither T, Wenzel C, Bräuer-Hartmann D, Wabitsch M, Behre HM. Inhibition of adipogenic differentiation of human SGBS preadipocytes by androgen-regulated microRNA miR-375. Mol Cell Endocrinol. 2015 Oct;414:177–85.

98 Krieg L, Schaffert A, Kern M, Landgraf K, Wabitsch M, Beck-Sickinger AG, et al. An MRM-Based Multiplexed Quantification Assay for Human Adipokines and Apolipoproteins. Molecules. 2020 Feb;25(4). DOI: 10.3390/molecules25040775

99 Kristóf E, Doan-Xuan QM, Sárvári AK, Klusóczki, Fischer-Posovszky P, Wabitsch M, et al. Clozapine modifies the differentiation program of human adipocytes inducing browning. Transl Psychiatry. 2016;6(11). DOI: 10.1038/tp.2016.230

100 Lahnalampi M, Heinäniemi M, Sinkkonen L, Wabitsch M, Carlberg C. Time-resolved expression profiling of the nuclear receptor superfamily in human adipogenesis. {PloS} One. 2010;5(9):e12991.

101 Lasa A, Schweiger M, Kotzbeck P, Churruca I, Simón E, Zechner R, et al. Resveratrol regulates lipolysis via adipose triglyceride lipase. J Nutr Biochem. 2012 Apr;23(4):379–84.

102 Laumen H, Skurk T, Hauner H. The HMG-CoA reductase inhibitor rosuvastatin inhibits plasminogen activator inhibitor-1 expression and secretion in human adipocytes. Atherosclerosis. 2008 Feb;196(2):565–73.

103 Leiherer A, Geiger K, Muendlein A, Drexel H. Hypoxia induces a HIF-1α dependent signaling cascade to make a complex metabolic switch in SGBS-adipocytes. Mol Cell Endocrinol. 2014 Mar;383(1–2):21–31.

104 Leiherer A, Stoemmer K, Muendlein A, Saely CH, Kinz E, Brandtner EM, et al. Quercetin impacts expression of metabolism-and obesity-associated genes in SGBS adipocytes. Nutrients. 2016 May;8(5). DOI: 10.3390/nu8050282

105 Li J, Daly E, Campioli E, Wabitsch M, Papadopoulos V. De novo synthesis of steroids and oxysterols in adipocytes. J Biol Chem. 2014 Jan;289(2):747–64.

106 Li S, Bouzar C, Cottet-Rousselle C, Zagotta I, Lamarche F, Wabitsch M, et al. Resveratrol inhibits lipogenesis of 3T3-L1 and SGBS cells by inhibition of insulin signaling and mitochondrial mass increase. Biochim Biophys Acta - Bioenerg. 2016 Jun;1857(6):643–52.

107 Limoge F, Faivre L, Gautier T, Petit JM, Gautier E, Masson D, et al. Insulin response dysregulation explains abnormal fat storage and increased risk of diabetes mellitus type 2 in Cohen Syndrome. Hum Mol Genet. 2015 Dec;24(23):6603–13.

108 Lin TY, Chiu CJ, Kuan CH, Chen FH, Shen YC, Wu CH, et al. IL-29 promoted obesity-induced inflammation and insulin resistance. Cell Mol Immunol. 2020 Apr;17(4):369–79.

109 Lo Re O, Maugeri A, Hruskova J, Jakubik J, Kucera J, Bienertova-Vasku J, et al. Obesity-induced nucleosome release predicts poor cardio-metabolic health. Clin Epigenetics. 2019 Dec;12(1). DOI: 10.1186/s13148-019-0797-8

110 Löffler D, Landgraf K, Körner A, Kratzsch J, Kirkby KC, Himmerich H. Modulation of triglyceride accumulation in adipocytes by psychopharmacological agents in vitro. J Psychiatr Res. 2016 Jan;72:37–42.

111 Ludewig AH, Klapper M, Wabitsch M, Döring F, Nitz I. Differential expression of alternative Acyl-CoA binding protein (ACBP) transcripts in an inducible human preadipocyte cell line. Horm Metab Res. 2011 Jun;43(6):440–2.

112 Luo X, Hutley LJ, Webster JA, Kim YH, Liu DF, Newell FS, et al. Identification of BMP and activin membrane-bound inhibitor (BAMBI) as a potent negative regulator of adipogenesis and modulator of autocrine/paracrine adipogenic factors. Diabetes. 2012 Jan;61(1):124–36.

113 Mack I, Belaiba RS, Djordjevic T, Görlach A, Hauner H, Bader BL. Functional analyses reveal the greater potency of preadipocytes compared with adipocytes as endothelial cell activator under normoxia, hypoxia, and TNFα exposure. Am J Physiol - Endocrinol Metab. 2009 Sep;297(3). DOI: 10.1152/ajpendo.90851.2008

114 Mandard S, Zandbergen F, Nguan ST, Escher P, Patsouris D, Koenig W, et al. The direct peroxisome proliferator-activated receptor target fasting-induced adipose factor (FIAF/PGAR/ANGPTL4) is present in blood plasma as a truncated protein that is increased by fenofibrate treatment. J Biol Chem. 2004 Aug;279(33):34411–20.

115 Mandrika I, Tilgase A, Petrovska R, Klovins J. Hydroxycarboxylic acid receptor ligands modulate proinflammatory cytokine expression in human macrophages and adipocytes without affecting adipose differentiation. Biol Pharm Bull. 2018;41(10):1574–80.

116 Massaro M, Scoditti E, Pellegrino M, Carluccio MA, Calabriso N, Wabitsch M, et al. Therapeutic potential of the dual peroxisome proliferator activated receptor (PPAR)α/γ agonist aleglitazar in attenuating TNF-α-mediated inflammation and insulin resistance in human adipocytes. Pharmacol Res. 2016 May;107:125–36.

117 Matthae S, May S, Hubersberger M, Hauner H, Skurk T. Protein normalization in different adipocyte models and dependence on cell size. Horm Metab Res. 2013;45(8):572–80.

118 McGillicuddy FC, Chiquoine EH, Hinkle CC, Kim RJ, Shah R, Roche HM, et al. Interferon γ attenuates insulin signaling, lipid storage, and differentiation in human adipocytes via activation of the JAK/STAT pathway. J Biol Chem. 2009;284(46):31936–44.

119 McInnes KJ, Brown KA, Hunger NI, Simpson ER. Regulation of LKB1 expression by sex hormones in adipocytes. Int J Obes. 2012 Jul;36(7):982–5.

120 McInnes KJ, Brown KA, Knower KC, Chand AL, Clyne CD, Simpson ER. Characterisation of aromatase expression in the human adipocyte cell line SGBS. Breast Cancer Res Treat. 2008 Dec;112(3):429–35.

121 Miehle F, Möller G, Cecil A, Lintelmann J, Wabitsch M, Tokarz J, et al. Lipidomic phenotyping reveals extensive lipid remodeling during adipogenesis in human adipocytes. Metabolites. 2020 Jun;10(6). DOI: 10.3390/metabo10060217

122 Mochalski P, Diem E, Unterkofler K, Mündlein A, Drexel H, Mayhew CA, et al. In vitro profiling of volatile organic compounds released by Simpson-Golabi-Behmel syndrome adipocytes. J Chromatogr B Anal Technol Biomed Life Sci. 2019 Jan;1104:256–61.

123 Molonia MS, Occhiuto C, Muscarà C, Speciale A, Bashllari R, Villarroya F, et al. Cyanidin-3-O-glucoside restores insulin signaling and reduces inflammation in hypertrophic adipocytes. Arch Biochem Biophys. 2020 Sep;691. DOI: 10.1016/j.abb.2020.108488

124 Molonia MS, Quesada-Lopez T, Speciale A, Muscarà C, Saija A, Villarroya F, et al. In Vitro Effects of Cyanidin-3-O-Glucoside on Inflammatory and Insulin-Sensitizing Genes in Human Adipocytes Exposed to Palmitic Acid. Chem Biodivers. 2021 Dec;18(12). DOI: 10.1002/cbdv.202100607

125 Mondal AK, Das SK, Baldini G, Chu WS, Sharma NK, Hackney OG, et al. Genotype and tissue-specific effects on alternative splicing of the transcription factor 7-like 2 gene in humans. J Clin Endocrinol Metab. 2010;95(3):1450–7.

126 Montanari T, Colitti M. Simpson–Golabi–Behmel syndrome human adipocytes reveal a changing phenotype throughout differentiation. Histochem Cell Biol. 2018 Jun;0(0):0.

127 Montt-Guevara MM, Finiguerra M, Marzi I, Fidecicchi T, Ferrari A, Genazzani AD, et al. D-Chiro-Inositol Regulates Insulin Signaling in Human Adipocytes. Front Endocrinol (Lausanne). 2021 Mar;12. DOI: 10.3389/fendo.2021.660815

128 Moreno-Navarrete JM, Escoté X, Ortega F, Serino M, Campbell M, Michalski MC, et al. A role for adipocyte-derived lipopolysaccharide-binding protein in inflammation- and obesity-associated adipose tissue dysfunction. Diabetologia. 2013 Nov;56(11):2524–37.

129 Moure R, Domingo P, Gallego-Escuredo JM, Villarroya J, Gutierrez MDM, Mateo MG, et al. Impact of elvitegravir on human adipocytes: Alterations in differentiation, gene expression and release of adipokines and cytokines. Antiviral Res. 2016 Aug;132:59–65.

130 Moure R, Domingo P, Villarroya J, Gasa L, Gallego-Escuredo JM, Quesada-López T, et al. Reciprocal effects of antiretroviral drugs used to treat HIV infection on the fibroblast growth factor 21/-klotho system. Antimicrob Agents Chemother. 2018 Jun;62(6). DOI: 10.1128/AAC.00029-18

131 Mracek T, Ding Q, Tzanavari T, Kos K, Pinkney J, Wilding J, et al. The adipokine zinc-α2-glycoprotein (ZAG) is downregulated with fat mass expansion in obesity. Clin Endocrinol (Oxf). 2010 Mar;72(3):334–41.

132 Murholm M, Isidor MS, Basse AL, Winther S, Sørensen C, Skovgaard-Petersen J, et al. Retinoic acid has different effects on {UCP}1 expression in mouse and human adipocytes. {BMC} cell Biol. 2013 Sep;14(1):41.

133 Muschet C, Möller G, Prehn C, de Angelis MH, Adamski J, Tokarz J. Removing the bottlenecks of cell culture metabolomics: fast normalization procedure, correlation of metabolites to cell number, and impact of the cell harvesting method. Metabolomics. 2016 Oct;12(10). DOI: 10.1007/s11306-016-1104-8

134 Mysore R, Zhou Y, Sädevirta S, Savolainen-Peltonen H, Nidhina Haridas PA, Soronen J, et al. MicroRNA-192* impairs adipocyte triglyceride storage. Biochim Biophys Acta - Mol Cell Biol Lipids. 2016 Apr;1861(4):342–51.

135 Nadra K, Medard J-J, Mul JD, Han G-S, Gres S, Pende M, et al. Cell Autonomous Lipin 1 Function Is Essential for Development and Maintenance of White and Brown Adipose Tissue. Mol Cell Biol. 2012 Dec;32(23):4794–810.

136 Nagel SA, Keuper M, Zagotta I, Enlund E, Ruperez AI, Debatin KM, et al. Up-regulation of Bcl-2 during adipogenesis mediates apoptosis resistance in human adipocytes. Mol Cell Endocrinol. 2014 Jan;382(1):368–76.

137 Nassiri I, Lombardo R, Lauria M, Morine MJ, Moyseos P, Varma V, et al. Systems view of adipogenesis via novel omics-driven and tissue-specific activity scoring of network functional modules. Sci Rep. 2016 Jul;6. DOI: 10.1038/srep28851

138 Newell FS, Su H, Tornqvist H, Whitehead JP, Prins JB, Hutley LJ, et al. Characterization of the transcriptional and functional effects of fibroblast growth factor-1 on human preadipocyte differentiation. FASEB J. 2006 Dec;20(14):2615–7.

139 Nixon M, Wake DJ, Livingstone DE, Stimson RH, Esteves CL, Seckl JR, et al. Salicylate downregulates 11β-HSD1 expression in adipose tissue in obese mice and in humans, mediating insulin sensitization. Diabetes. 2012 Apr;61(4):790–6.

140 Noer A, Serensen AL, Boquest AC, Collas P. Stable CpG hypomethylation of adipogenic promoters in freshly isolated, cultured, and differentiated mesenchymal stem cells from adipose tissue. Mol Biol Cell. 2006 Aug;17(8):3543–56.

141 Ogunkolade BW, Adaikalakoteswari A, Cardoso SR, Lowe R, Patel N, Rakyan V, et al. An integrative epi-transcriptomic approach identifies the human cartilage chitinase 3-like protein 2 (CHI3L2) as a potential mediator of B12 deficiency in adipocytes. Epigenetics. 2021 Nov;1–15.

142 O’Hara A, Lim F-L, Mazzatti DJ, Trayhurn P. Microarray analysis identifies matrix metalloproteinases (MMPs) as key genes whose expression is up-regulated in human adipocytes by macrophage-conditioned medium. Pflugers Arch. 2009 Oct;458(6):1103–14.

143 Osorio-Conles O, Guitart M, Chacón MR, Maymo-Masip E, Moreno-Navarrete JM, Montori-Grau M, et al. Plasma PTX3 protein levels inversely correlate with insulin secretion and obesity, whereas visceral adipose tissue PTX3 gene expression is increased in obesity. Am J Physiol - Endocrinol Metab. 2011 Dec;301(6). DOI: 10.1152/ajpendo.00163.2011

144 Osorio-Conles Ó, Guitart M, Moreno-Navarrete JM, Escoté X, Duran X, Fernandez-Real JM, et al. Adipose tissue and serum CCDC80 in obesity and its association with related metabolic disease. Mol Med. 2017;23:225–34.

145 Palominos MM, Dünner NH, Wabitsch M, Rojas C V. Angiotensin II directly impairs adipogenic differentiation of human preadipose cells. Mol Cell Biochem. 2015 Oct;408(1–2):115–22.

146 Patsouris D, Mandard S, Voshol PJ, Escher P, Tan NS, Havekes LM, et al. PPARα governs glycerol metabolism. J Clin Invest. 2004 Jul;114(1):94–103.

147 Poetke M, Jamil B, Müller U, Berlien HP. Diffuse neonatal hemangiomatosis associated with Simpson-Golabi-Behmel syndrome: A case report. Eur J Pediatr Surg. 2002;12(1):59–62.

148 Price NL, Holtrup B, Kwei SL, Wabitsch M, Rodeheffer M, Bianchini L, et al. SREBP-1c/MicroRNA 33b Genomic Loci Control Adipocyte Differentiation. Mol Cell Biol. 2016 Apr;36(7):1180–93.

149 Qiao Q, Bouwman FG, Renes J, Mariman ECM. An in vitro model for hypertrophic adipocytes: Time‐dependent adipocyte proteome and secretome changes under high glucose and high insulin conditions. J Cell Mol Med. 2020 Jul;jcmm.15497.

150 Qiao Q, Bouwman FG, Van Baak MA, Renes J, Mariman ECM. Glucose restriction plus refeeding in vitro induce changes of the human adipocyte secretome with an impact on complement factors and cathepsins. Int J Mol Sci. 2019 Aug;20(16). DOI: 10.3390/ijms20164055

151 Qiao Q, Bouwman FG, van Baak MA, Roumans NJT, Vink RG, Coort SLM, et al. Adipocyte abundances of CES1, CRYAB, ENO1 and GANAB are modified in-vitro by glucose restriction and are associated with cellular remodelling during weight regain. Adipocyte. 2019 Jan;8(1):190–200.

152 Quarta S, Scoditti E, Carluccio MA, Calabriso N, Santarpino G, Damiano F, et al. Coffee bioactive n-methylpyridinium attenuates tumor necrosis factor (Tnf)-α-mediated insulin resistance and inflammation in human adipocytes. Biomolecules. 2021 Oct;11(10). DOI: 10.3390/biom11101545

153 Rae-Whitcombe SM, Kennedy D, Voyles M, Thompson MP. Regulation of the promoter region of the human adiponutrin/PNPLA3 gene by glucose and insulin. Biochem Biophys Res Commun. 2010 Nov;402(4):767–72.

154 Renes J, Rosenow A, Roumans N, Noben JP, Mariman ECM. Calorie restriction-induced changes in the secretome of human adipocytes, comparison with resveratrol-induced secretome effects. Biochim Biophys Acta - Proteins Proteomics. 2014;1844(9):1511–22.

155 Revollo JR, Körner A, Mills KF, Satoh A, Wang T, Garten A, et al. Nampt/PBEF/Visfatin Regulates Insulin Secretion in β Cells as a Systemic NAD Biosynthetic Enzyme. Cell Metab. 2007 Nov;6(5):363–75.

156 Rockstroh D, Löffler D, Kiess W, Landgraf K, Körner A. Regulation of human adipogenesis by miR125b-5p. Adipocyte. 2016 Jul;5(3):283–97.

157 Roos J, Dahlhaus M, Funcke JB, Kustermann M, Strauss G, Halbgebauer D, et al. miR-146a regulates insulin sensitivity via NPR3. Cell Mol Life Sci. 2021 Mar;78(6):2987–3003.

158 Roos J, Enlund E, Funcke J-BB, Tews D, Holzmann K, Debatin K-MM, et al. miR-146a-mediated suppression of the inflammatory response in human adipocytes. Sci Rep. 2016 Dec;6(1):38339.

159 Rose FJ, Webster J, Barry JB, Phillips LK, Richards AA, Whitehead JP. Synergistic effects of ascorbic acid and thiazolidinedione on secretion of high molecular weight adiponectin from human adipocytes. Diabetes, Obes Metab. 2010;12(12):1084–9.

160 Rosenow A, Arrey TN, Bouwman FG, Noben J-PP, Wabitsch M, Mariman ECMM, et al. Identification of novel human adipocyte secreted proteins by using {SGBS} cells. J Proteome Res. 2010 Oct;9(10):5389–401.

161 Rosenow A, Noben JP, Bouwman FG, Mariman ECM, Renes J. Hypoxia-mimetic effects in the secretome of human preadipocytes and adipocytes. Biochim Biophys Acta - Proteins Proteomics. 2013;1834(12):2761–71.

162 Rosenow A, Noben JP, Jocken J, Kallendrusch S, Fischer-Posovszky P, Mariman ECM, et al. Resveratrol-induced changes of the human adipocyte secretion profile. J Proteome Res. 2012 Sep;11(9):4733–43.

163 Rossi A, Eid M, Dodgson J, Davies G, Musial B, Wabitsch M, et al. In vitro characterization of the effects of chronic insulin stimulation in mouse 3T3-L1 and human SGBS adipocytes. Adipocyte. 2020;9(1):415–26.

164 Roumans NJT, Camps SG, Renes J, Bouwman FG, Westerterp KR, Mariman ECM. Weight loss-induced stress in subcutaneous adipose tissue is related to weight regain. Br J Nutr. 2016 Jan;115(5):913–20.

165 Ryynänen J, Neme A, Tuomainen TP, Virtanen JK, Voutilainen S, Nurmi T, et al. Changes in vitamin D target gene expression in adipose tissue monitor the vitamin D response of human individuals. Mol Nutr Food Res. 2014;58(10):2036–45.

166 Sæther T, Paulsen SM, Tungen JE, Vik A, Aursnes M, Holen T, et al. Synthesis and biological evaluations of marine oxohexadecenoic acids: PPARα/γ dual agonism and anti-diabetic target gene effects. Eur J Med Chem. 2018 Jul;155:736–53.

167 Schaedlich K, Beier L-SS, Kolbe J, Wabitsch M, Ernst J. Pro-inflammatory effects of DEHP in SGBS-derived adipocytes and THP-1 macrophages. Sci Rep. 2021 Dec;11(1). DOI: 10.1038/s41598-021-85119-3

168 Schaedlich K, Gebauer S, Hunger L, Beier LS, Koch HM, Wabitsch M, et al. DEHP deregulates adipokine levels and impairs fatty acid storage in human SGBS-adipocytes. Sci Rep. 2018 Dec;8(1). DOI: 10.1038/s41598-018-21800-4

169 Schaffert A, Krieg L, Weiner J, Schlichting R, Ueberham E, Karkossa I, et al. Alternatives for the worse: Molecular insights into adverse effects of bisphenol a and substitutes during human adipocyte differentiation. Environ Int. 2021 Nov;156:106730.

170 Schlottmann I, Ehrhart-Bornstein M, Wabitsch M, Bornstein SR, Lamounier-Zepter V. Calcium-dependent release of adipocyte fatty acid binding protein from human adipocytes. Int J Obes. 2014 Sep;38(9):1221–7.

171 Schmidt SF, Jørgensen M, Chen Y, Nielsen R, Sandelin A, Mandrup S. Cross species comparison of C/EBPα and PPARγ profiles in mouse and human adipocytes reveals interdependent retention of binding sites. BMC Genomics. 2011 Mar;12. DOI: 10.1186/1471-2164-12-152

172 Schweiger M, Paar M, Eder C, Brandis J, Moser E, Gorkiewicz G, et al. G0/G1 switch gene-2 regulates human adipocyte lipolysis by affecting activity and localization of adipose triglyceride lipase. J Lipid Res. 2012 Nov;53(11):2307–17.

173 Scoditti E, Carpi S, Massaro M, Pellegrino M, Polini B, Carluccio MA, et al. Hydroxytyrosol modulates adipocyte gene and mirna expression under inflammatory condition. Nutrients. 2019 Oct;11(10). DOI: 10.3390/nu11102493

174 Scoditti E, Massaro M, Carluccio MA, Pellegrino M, Wabitsch M, Calabriso N, et al. Additive regulation of adiponectin expression by the mediterranean diet olive oil components oleic acid and hydroxytyrosol in human adipocytes. PLoS One. 2015 Jun;10(6). DOI: 10.1371/journal.pone.0128218

175 Senol-Cosar O, Flach RJR, Distefano M, Chawla A, Nicoloro S, Straubhaar J, et al. Tenomodulin promotes human adipocyte differentiation and beneficial visceral adipose tissue expansion. Nat Commun. 2016 Feb;7. DOI: 10.1038/ncomms10686

176 Serrano-Marco L, Chacón MR, Maymó-Masip E, Barroso E, Salvadó L, Wabitsch M, et al. TNF-α inhibits PPARβ/δ activity and SIRT1 expression through NF-κB in human adipocytes. Biochim Biophys Acta - Mol Cell Biol Lipids. 2012 Sep;1821(9):1177–85.

177 Sharma NK, Key CCC, Civelek M, Wabitsch M, Comeau ME, Langefeld CD, et al. Genetic regulation of Enoyl-CoA hydratase domain-containing 3 in adipose tissue determines insulin sensitivity in African Americans and Europeans. Diabetes. American Diabetes Association Inc.; 2019; pp 1508–22.

178 Sharma N, Varma V, Ma L, Hasstedt S, Das S. Obesity Associated Modulation of miRNA and Co-Regulated Target Transcripts in Human Adipose Tissue of Non-Diabetic Subjects. MicroRNA. 2016 Jan;4(3):194–204.

179 Shi X, Shi W, Li Q, Song B, Wan M, Bai S, et al. A glucocorticoid-induced leucine-zipper protein, GILZ, inhibits adipogenesis of mesenchymal cells. EMBO Rep. 2003 Apr;4(4):374–80.

180 Shreder K, Rapp F, Tsoukala I, Rzeznik V, Wabitsch M, Fischer-Posovszky P, et al. Impact of x-ray exposure on the proliferation and differentiation of human pre-adipocytes. Int J Mol Sci. 2018 Sep;19(9). DOI: 10.3390/ijms19092717

181 Simon MF, Daviaud D, Pradère JP, Grès S, Guigné C, Wabitsch M, et al. Lysophosphatidic acid inhibits adipocyte differentiation via lysophosphatidic acid 1 receptor-dependent down-regulation of peroxisome proliferator-activated receptor γ2. J Biol Chem. 2005 Apr;280(15):14656–62.

182 Soccio RE, Tuteja G, Everett LJ, Li Z, Lazar MA, Kaestner KH. Species-specific strategies underlying conserved functions of metabolic transcription factors. Mol Endocrinol. 2011 Apr;25(4):694–706.

183 Stadion M, Schwerbel K, Graja A, Baumeier C, Rödiger M, Jonas W, et al. Increased Ifi202b/IFI16 expression stimulates adipogenesis in mice and humans. Diabetologia. 2018 May;61(5):1167–79.

184 Standeven KF, Hess K, Carter AM, Rice GI, Cordell PA, Balmforth AJ, et al. Neprilysin, obesity and the metabolic syndrome. Int J Obes. 2011 Aug;35(8):1031–40.

185 Stienstra R, Joosten LAB, Koenen T, Van Tits B, van Diepen JA, Van Den Berg SAA, et al. The inflammasome-mediated caspase-1 activation controls adipocyte differentiation and insulin sensitivity. Cell Metab. 2010 Dec;12(6):593–605.

186 Szatmári-Tóth M, Shaw A, Csomós I, Mocsár G, Fischer-Posovszky P, Wabitsch M, et al. Thermogenic activation downregulates high mitophagy rate in human masked and mature beige adipocytes. Int J Mol Sci. 2020 Sep;21(18):1–21.

187 Tang J, Pulliam N, Ozeş A, Buechlein A, Ding N, Keer H, et al. Epigenetic targeting of adipocytes inhibits high-grade serous ovarian cancer cell migration and invasion. Mol Cancer Res. 2018 Aug;16(8):1226–40.

188 Tanigawa Y, Li J, Justesen JM, Horn H, Aguirre M, DeBoever C, et al. Components of genetic associations across 2,138 phenotypes in the UK Biobank highlight adipocyte biology. Nat Commun. 2019 Dec;10(1). DOI: 10.1038/s41467-019-11953-9

189 Tews D, Fromme T, Keuper M, Hofmann SM, Debatin KM, Klingenspor M, et al. Teneurin-2 (TENM2) deficiency induces UCP1 expression in differentiating human fat cells. Mol Cell Endocrinol. 2017 Mar;443:106–13.

190 Tews D, Pula T, Funcke JB, Jastroch M, Keuper M, Debatin KM, et al. Elevated UCP1 levels are sufficient to improve glucose uptake in human white adipocytes. Redox Biol. 2019 Sep;26:101286.

191 Tews D, Fischer-Posovszky P, Wabitsch M. Regulation of FTO and FTM Expression During Human Preadipocyte Differentiation. Horm Metab Res. 2011 Jan;43(01):17–21.

192 Tian Y, Kijlstra A, Renes J, Wabitsch M, Webers CAB, Berendschot TTJM. Lutein Leads to a Decrease of Factor D Secretion by Cultured Mature Human Adipocytes. J Ophthalmol. 2015;2015. DOI: 10.1155/2015/430741

193 Tiller G, Laumen H, Fischer-Posovszky P, Finck A, Skurk T, Keuper M, et al. {LIGHT} ({TNFSF}14) inhibits adipose differentiation without affecting adipocyte metabolism. Int J Obes (Lond). 2011 Feb;35(2):208–16.

194 Tomabechi Y, Tsuruta T, Saito S, Wabitsch M, Sonoyama K. Extra-adrenal glucocorticoids contribute to the postprandial increase of circulating leptin in mice. J Cell Commun Signal. 2018 Jun;12(2):433–9.

195 Tréguer K, Dusaulcy R, Grès S, Wanecq E, Valet P, Saulnier-Blache JS. Influence of secreted factors from human adipose tissue on glucose utilization and proinflammatory reaction. J Physiol Biochem. 2013 Sep;69(3):625–32.

196 Vaittinen M, Kaminska D, Kakela P, Eskelinen M, Kolehmainen M, Pihlajamaki J, et al. Downregulation of CPPED1 Expression Improves Glucose Metabolism In Vitro in Adipocytes. Diabetes. 2013 Nov;62(11):3747–50.

197 Vaittinen M, Kolehmainen M, Rydén M, Eskelinen M, Wabitsch M, Pihlajamäki J, et al. MFAP5 is related to obesity-associated adipose tissue and extracellular matrix remodeling and inflammation. Obesity. 2015 Jul;23(7):1371–8.

198 Varinli H, Osmond-Mcleod MJ, Molloy PL, Vallotton P. LipiD-QuanT: A novel method to quantify lipid accumulation in live cells. J Lipid Res. 2015 Nov;56(11):2206–16.

199 Varma V, Boros LG, Nolen GT, Chang C-W, Wabitsch M, Beger RD, et al. Metabolic fate of fructose in human adipocytes: a targeted 13C tracer fate association study. Metabolomics. 2015 Jun;11(3):529–44.

200 Varma V, Boros LG, Nolen GT, Chang C-W, Wabitsch M, Beger RD, et al. Fructose Alters Intermediary Metabolism of Glucose in Human Adipocytes and Diverts Glucose to Serine Oxidation in the One-Carbon Cycle Energy Producing Pathway. Metabolites. 2015 Jun;5(2):364–85.

201 Vasileva LV, Savova MS, Tews D, Wabitsch M, Georgiev MI. Rosmarinic acid attenuates obesity and obesity-related inflammation in human adipocytes. Food Chem Toxicol. 2021;149(January):112002.

202 Velazquez-Villegas LA, Perino A, Lemos V, Zietak M, Nomura M, Pols TWH, et al. TGR5 signalling promotes mitochondrial fission and beige remodelling of white adipose tissue. Nat Commun. 2018 Dec;9(1). DOI: 10.1038/s41467-017-02068-0

203 Verstraeten VLRM, Renes J, Ramaekers FCS, Kamps M, Kuijpers HJ, Verheyen F, et al. Reorganization of the nuclear lamina and cytoskeleton in adipogenesis. Histochem Cell Biol. 2011 Mar;135(3):251–61.

204 Wabitsch M, Brenner RE, Melzner I, Braun M, Möller P, Heinze E, et al. Characterization of a human preadipocyte cell strain with high capacity for adipose differentiation. Int J Obes Relat Metab Disord. 2001 Jan;25(1):8–15.

205 Weaver RE, Donnelly D, Wabitsch M, Grant PJ, Balmforth AJ. Functional expression of glucose-dependent insulinotropic polypeptide receptors is coupled to differentiation in a human adipocyte model. Int J Obes. 2008 Nov;32(11):1705–11.

206 Weber S, Salabei JK, Möller G, Kremmer E, Bhatnagar A, Adamski J, et al. Aldo-keto Reductase 1B15 (AKR1B15): A mitochondrial human aldo-keto reductase with activity toward steroids and 3-keto-acyl-CoA conjugates. J Biol Chem. 2015 Mar;290(10):6531–45.

207 Webster JA, Yang Z, Kim YH, Loo D, Mosa RM, Li H, et al. Collagen beta (1-O) galactosyltransferase 1 (GLT25D1) is required for the secretion of high molecular weight adiponectin and affects lipid accumulation. Biosci Rep. 2017 Jun;37(3). DOI: 10.1042/BSR20170105

208 Wei Y, Puzhko S, Wabitsch M, Goodyer CG. Structure and activity of the human growth hormone receptor ({hGHR}) gene V2 promoter. Mol Endocrinol. 2009 Mar;23(3):360–72.

209 Wei Y, Puzhko S, Wabitsch M, Goodyer CG. Transcriptional regulation of the human growth hormone receptor (hGHR) gene V2 promoter by transcriptional activators and repressor. Mol Endocrinol. 2009 Mar;23(3):373–87.

210 Wei Y, Rhani Z, Goodyer CG. Characterization of growth hormone receptor messenger ribonucleic acid variants in human adipocytes. J Clin Endocrinol Metab. 2006 May;91(5):1901–8.

211 Wentworth JM, Zhang JG, Bandala-Sanchez E, Naselli G, Liu R, Ritchie M, et al. Interferon-gamma released from omental adipose tissue of insulin-resistant humans alters adipocyte phenotype and impairs response to insulin and adiponectin release. Int J Obes. 2017 Dec;41(12):1782–9.

212 Wittrisch S, Klöting N, Mörl K, Chakaroun R, Blüher M, Beck-Sickinger AG. NPY1R-targeted peptide-mediated delivery of a dual PPARα/γ agonist to adipocytes enhances adipogenesis and prevents diabetes progression. Mol Metab. 2020 Jan;31:163–80.

213 Wood IS, Wang B, Trayhurn P. IL-33, a recently identified interleukin-1 gene family member, is expressed in human adipocytes. Biochem Biophys Res Commun. 2009 Jun;384(1):105–9.

214 Wood IS, Hunter L, Trayhurn P. Expression of Class III facilitative glucose transporter genes (GLUT-10 and GLUT-12) in mouse and human adipose tissues. Biochem Biophys Res Commun. 2003 Aug;308(1):43–9.

215 Wu H, Pula T, Tews D, Amri EZ, Debatin KM, Wabitsch M, et al. Microrna-27a-3p but not-5p is a crucial mediator of human adipogenesis. Cells. 2021 Nov;10(11). DOI: 10.3390/cells10113205

216 Wu X, Sakharkar MK, Wabitsch M, Yang J. Effects of sphingosine-1-phosphate on cell viability, differentiation, and gene expression of adipocytes. Int J Mol Sci. 2020 Dec;21(23):1–16.

217 Xu H, Barnes GT, Yang Q, Tan G, Yang D, Chou CJ, et al. Chronic inflammation in fat plays a crucial role in the development of obesity-related insulin resistance. J Clin Invest. 2003 Dec;112(12):1821–30.

218 Yao-Borengasser A, Rassouli N, Varma V, Bodles AM, Rasouli N, Unal R, et al. Stearoyl-coenzyme A desaturase 1 gene expression increases after pioglitazone treatment and is associated with peroxisomal proliferator-activated receptor-γ responsiveness. J Clin Endocrinol Metab. 2008;93(11):4431–9.

219 Yeo CR, Agrawal M, Hoon S, Shabbir A, Shrivastava MK, Huang S, et al. Reply to: ‘Browning capabilities of human primary adipose-derived stromal cells compared to SGBS cells.’ Sci Rep. 2020 Dec;10(1). DOI: 10.1038/s41598-020-64706-w

220 Yeo CR, Agrawal M, Hoon S, Shabbir A, Shrivastava MK, Huang S, et al. SGBS cells as a model of human adipocyte browning: A comprehensive comparative study with primary human white subcutaneous adipocytes. Sci Rep. 2017 Dec;7(1):4031.

221 Zagotta I, Dimova EY, Funcke JB, Wabitsch M, Kietzmann T, Fischer-Posovszky P. Resveratrol suppresses PAI-1 gene expression in a human in vitro model of inflamed adipose tissue. Oxid Med Cell Longev. 2013 DOI: 10.1155/2013/793525

222 Zandbergen F, Mandard S, Escher P, Tan NS, Patsouris D, Jatkoe T, et al. The G0/G1 switch gene 2 is a novel PPAR target gene. Biochem J. 2005 Dec;392(2):313–24.

223 Zhang Y, McGillicuddy FC, Hinkle CC, O’Neill S, Glick JM, Rothblat GH, et al. Adipocyte modulation of high-density lipoprotein cholesterol. Circulation. 2010 Mar;121(11):1347–55.

224 Zhao J, Deliard S, Aziz AR, Grant SF. Expression analyses of the genes harbored by the type 2 diabetes and pediatric BMI associated locus on 10q23. BMC Med Genet. 2012 Dec;13(1):89.

225 Zhao J, Gray SG, Wabitsch M, Greene CM, Lawless MW. The therapeutic properties of resminostat for hepatocellular carcinoma. Oncoscience. 2018 Jun;5(5–6):196–208.

226 Zhou Y, Robciuc MR, Wabitsch M, Juuti A, Leivonen M, Ehnholm C, et al. OSBP-Related Proteins (ORPs) in Human Adipose Depots and Cultured Adipocytes: Evidence for Impacts on the Adipocyte Phenotype. PLoS One. 2012 Sep;7(9). DOI: 10.1371/journal.pone.0045352

227 Zoller V, Funcke JB, Keuper M, El Hay MA, Debatin KM, Wabitsch M, et al. TRAIL (TNF-related apoptosis-inducing ligand) inhibits human adipocyte differentiation via caspase-mediated downregulation of adipogenic transcription factors. Cell Death Dis. 2016 Oct;7(10). DOI: 10.1038/cddis.2016.286

228 Zoller V, Funcke JB, Roos J, Dahlhaus M, Abd El Hay M, Holzmann K, et al. Trail (TNF-related apoptosis-inducing ligand) induces an inflammatory response in human adipocytes. Sci Rep. 2017 Dec;7(1). DOI: 10.1038/s41598-017-05932-7
